# DO SLEEP QUALITY AND SOCIAL COHESION MEDIATE ASSOCIATION BETWEEN NEIGHBORHOOD DISORDER AND COGNITIVE FUNCTIONING?

**DOI:** 10.1093/geroni/igad104.0505

**Published:** 2023-12-21

**Authors:** Darlingtina Esiaka, Elizabeth Luth

**Affiliations:** University of Kentucky, Lexington, Kentucky, United States; Rutgers University, New Brunswick, New Jersey, United States

## Abstract

Daily exposure to neighborhoods with high levels of disorder, such as public drinking, graffiti, and vandalism, has been linked to worse cognitive functioning and a higher risk for dementia in older adults. The present study examined the association between neighborhood disorder and cognitive functioning. This study also examined sleep quality and social cohesion as potential mediators of this association using Hayes’ (2013) Macro Process via bootstrapping method. The sample included cross-sectional data for 2,529 older adults (1,115 men and 1,482 women, age 70+) using the 2021 National Health and Aging Trends Study. Neighborhood disorder was measured as a 3-item scale capturing physical neighborhood characteristics completed by the interviewer (e.g., trash on sidewalks). The social cohesion scale measured participant agreement with 3 questions about their community (e.g., “People can be trusted”). The sleep quality scale measured participant responses to 3 questions about their sleep in the last month (e.g., taking 30+ minutes to fall asleep). Cognitive functioning was assessed using performance on tasks evaluating memory, orientation, and executive functioning. Results showed greater neighborhood disorder was associated with worse performance on cognitive tasks (B=-0.417; p<0.001). Results of mediation testing indicated that social cohesion was a significant mediator (B=0.224; p<0.0001). Though sleep quality was not a mediator, better sleep quality was associated with better cognitive functioning (B=0.053; p<0.05). Understanding the contribution of neighborhood disorder, sleep quality, and social cohesion to cognitive functioning will inform neighborhood-level interventions that can buffer cognitive decline in older adults aging in place.

